# Association between Lifetime Exposure to Inorganic Arsenic in Drinking Water and Coronary Heart Disease in Colorado Residents

**DOI:** 10.1289/ehp.1307839

**Published:** 2014-10-28

**Authors:** Katherine A. James, Tim Byers, John E. Hokanson, Jaymie R. Meliker, Gary O. Zerbe, Julie A. Marshall

**Affiliations:** 1 Colorado School of Public Health, University of Colorado Denver, Aurora, Colorado, USA; 2 Department of Preventive Medicine, State University of New York, Stony Brook, New York, USA

## Abstract

Background: Chronic diseases, including coronary heart disease (CHD), have been associated with ingestion of drinking water with high levels of inorganic arsenic (> 1,000 μg/L). However, associations have been inconclusive in populations with lower levels (< 100 μg/L) of inorganic arsenic exposure.

Objectives: We conducted a case-cohort study based on individual estimates of lifetime arsenic exposure to examine the relationship between chronic low-level arsenic exposure and risk of CHD.

Methods: This study included 555 participants with 96 CHD events diagnosed between 1984 and 1998 for which individual lifetime arsenic exposure estimates were determined using data from structured interviews and secondary data sources to determine lifetime residence, which was linked to a geospatial model of arsenic concentrations in drinking water. These lifetime arsenic exposure estimates were correlated with historically collected urinary arsenic concentrations. A Cox proportional-hazards model with time-dependent CHD risk factors was used to assess the association between time-weighted average (TWA) lifetime exposure to low-level inorganic arsenic in drinking water and incident CHD.

Results: We estimated a positive association between low-level inorganic arsenic exposure and CHD risk [hazard ratio (HR): = 1.38, 95% CI: 1.09, 1.78] per 15 μg/L while adjusting for age, sex, first-degree family history of CHD, and serum low-density lipoprotein levels. The risk of CHD increased monotonically with increasing TWAs for inorganic arsenic exposure in water relative to < 20 μg/L (HR = 1.2, 95% CI: 0.6, 2.2 for 20–30 μg/L; HR = 2.2; 95% CI: 1.2, 4.0 for 30–45 μg/L; and HR = 3, 95% CI: 1.1, 9.1 for 45–88 μg/L).

Conclusions: Lifetime exposure to low-level inorganic arsenic in drinking water was associated with increased risk for CHD in this population.

Citation: James KA, Byers T, Hokanson JE, Meliker JR, Zerbe GO, Marshall JA. 2015. Association between lifetime exposure to inorganic arsenic in drinking water and coronary heart disease in Colorado residents. Environ Health Perspect 123:128–134; http://dx.doi.org/10.1289/ehp.1307839

## Introduction

Nonoccupational exposure to inorganic arsenic occurs mainly through drinking contaminated water [U.S. Environmental Protection Agency (EPA) 1988]. In recent decades, research has identified a relationship between exposure to high concentrations of inorganic arsenic in drinking water and the risk of coronary heart disease (CHD); however, the risk at lower levels is ambiguous. Studies from Asia, where water concentrations of inorganic arsenic can be > 1,000 μg/L, have reported inorganic arsenic in drinking water to be associated with ischemic heart disease and carotid atherosclerosis (Chen CJ et al. 1996; Tseng et al. 2003; Wang et al. 2002), hypertension (Chen CJ et al. 1995, 2007; Chen Y et al. 2006a; Rahman et al. 1999), and intermediate outcomes associated with CHD including carotid artery intimal–medial thickness (Chen Y et al. 2006b) and ECG changes (Wang et al. 2010).

An association of cardiovascular risk with low-level arsenic exposure in drinking water (< 100 μg/L) has been suggested by recent studies (Chen Y et al. 2011; Moon et al. 2013). In a study by Chen Y et al. (2011), results suggested a higher cardiovascular mortality rate with exposure to drinking water arsenic concentrations > 12 μg/L, and an increasing trend in hazard ratios (HRs) with increasing arsenic exposure (log rank trend test = 0.0019) while controlling for known CHD risk factors. Positive associations were reported by other studies with similar exposure levels (< 100 μg/L) (Medrano et al. 2010; Sohel et al. 2009). These findings suggest that increased risk for cardiovascular disease occurs at levels similar to concentrations found in drinking water in areas of the United States.

In the United States, where arsenic concentrations are generally < 100 μg/L, ecologic studies (Engel and Smith 1994; Engel et al. 1994; Lewis et al. 1999; Meliker et al. 2007; Zierold et al. 2004) and review articles (Navas-Acien et al. 2005; Wang et al. 2007) have suggested a possible association of drinking-water arsenic with CHD, hypertension, and carotid intimal thickness. However, it has been only recently that chronic exposure to low to moderate levels inorganic arsenic in drinking water has been investigated as an independent risk factor for cardiovascular diseases in a prospective study. Moon et al. (2013) reported an association between urinary arsenic concentrations and coronary heart disease [HR = 1.16; 95% confidence interval (CI): 1.03, 1.30 per 9.9 μg/g adjusted for CHD risk factors] in U.S. American Indian communities. These findings in the Strong Heart Study (Moon et al. 2013) were the first to prospectively assess low- to moderate-level inorganic arsenic exposure in urine with cardiovascular disease at a community level; however, the study was limited in assessing exposure at the individual level. Future research that prospectively follows a cohort representative of U.S. communities with individual-level exposure assessment is necessary to further substantiate the association between inorganic arsenic exposure in drinking water and cardiovascular disease and elucidate the dose–response curve.

## Methods

We investigated the relationship between lifetime inorganic arsenic exposures and the risk of incident CHD using a case-cohort design within the San Luis Valley Diabetes Study (SLVDS). SLVDS is a population-based prospective study conducted from 1984 through 1998 in Alamosa and Conejos counties of south central Colorado. The study investigated the risk factors for diabetes mellitus (DM) and other related chronic diseases in Hispanic and non-Hispanic whites 20–74 years old. SLVDS data collection methods and participant recruitment have been described elsewhere (Hamman et al. 1989). In brief, researchers collected clinical, behavioral, and demographic data and diagnostic assessments including diagnoses of CHD from 1984 through 1988 (Hamman et al. 1989). Participants were then invited to attend follow-up visits every 4 years through 1998 to update their behavioral, demographic, and clinical assessments, along with an additional set of assessments on participants with impaired glucose tolerance at the initial visit. All participants were followed between clinic visits with telephone interviews and searches of vital statistics records to track vital status and identify underlying cause of death, where applicable (Hokanson et al. 2003). This cohort is stable, with a 98% follow-up of study participants through 1998 (Hokanson et al. 2003).

There were 1,297 SLVDS participants with no known CHD events or a diagnosis of DM before the baseline visit. Participants with a documented refusal for re-contact in SLVDS (*n* = 361) were excluded, leaving 936 participants eligible for this study. Cases of CHD included all eligible participants with a documented CHD event between their baseline visit and 1998. A CHD event was defined as any of the following: myocardial infarction, angioplasty, and death due to acute, subacute, or chronic ischemic heart disease [ICD-9 (*International Classification of Diseases, 9th Revision*) codes 410–414]. Potential CHD events were identified through self-report on yearly follow-up phone calls, obituary monitoring, and death certificate searches (Swenson et al. 2001). The medical records of identified CHD events were reviewed by a three-member committee of medical physicians for case confirmation.

The subcohort for estimating person-years of risk was randomly selected from the eligible participants without a previous diagnosis of CHD at the time of initial enrollment. The sample size of the subcohort was determined in Pass® software (www.ncss.com/software/pass/) based on recent research to estimate effect size (a relative risk of 1.4 for an increased risk > 10 μg/L) (Zierold et al. 2004) with alpha = 0.05 and power of 80%. The subcohort included 533 randomly selected participants, of whom 74 were incident CHD cases. The remaining 22 incident CHD cases not selected were added to the subcohort for a total of 555 participants in the CHD case-cohort study. Within the 555 study subjects, we had 64% (*n* = 357) participation rate, 33% (*n* = 189; 30% noncases and 46% cases) unable to locate, and 3% (*n* = 19) who refused participation.

*Estimating arsenic exposures*. Residential history (determined from structured interviews or secondary data sources) was linked to a geospatial model of predicted inorganic arsenic in groundwater to reconstruct annual estimated exposure to inorganic arsenic in drinking water over each participant’s lifetime. The use of inorganic arsenic levels in residential drinking water to assess exposure was supported by findings from our research that correlated annual predicted arsenic exposure estimates with temporally concurrent speciated inorganic arsenic concentrations in historically collected samples. Between 2006 and 2008, study subjects or next of kin of deceased subjects as designated in SLVDS (14.2%) were contacted by mail with information about the study, followed by a call to set up an appointment for an interview and water sample collection. During the interviews (*n* = 357; 64%) we collected data about past residences, past workplace/school locations, and history of drinking-water consumption at each location. For each location, data included addresses, residence dates, water source (well or public), water treatment device (if yes, type, model number), number of glasses of water consumed per day (nonbottled water), number of glasses of beverages made with water (e.g., coffee, tea, juice), whether they typically cooked with water from the tap in the home, and whether they had a vegetable garden (if yes, which vegetables). For subjects or next of kin who were not able to be located for an interview (*n* = 189, 33%), we used triangulation methods incorporating records from the county assessor’s office and SLVDS tracking database to reconstruct residential history. In brief, 189 participants were not interviewed (*n* = 2,023 person-years); therefore, the SLVDS contact tracking database was used to determine residence history back to 1975 and earlier for participants who reported living at the residence listed in 1975, before 1975. This left 914 person-years across 98 subjects still missing residential history who therefore were further investigated in county clerk records. In the end, there were 18 subjects with partial residential history (before 1975) (*n* = 126 person-years) with missing residential locations, and these were assigned the mean value for the last known city of residence.

Drinking-water samples were collected from the residential kitchen tap at time of interview and analyzed by the chemistry laboratory of the Colorado Department of Public Health and Environment using standard ion chromatography and inductively coupled plasma mass spectrometry with a detection limit of 1 μg/L. Samples with arsenic concentrations below the detection limit were given a value of half of the detection limit similar to other studies (Ayotte et al. 2006). Samples collected from private wells were assigned geographic coordinates using a global positioning system (GPS) unit (*n* = 248). Elsewhere we have provided methods for determining and validating the temporal and spatial variability of inorganic arsenic in groundwater in the SLV (James et al. 2013). In brief, findings indicate that naturally occurring inorganic arsenic concentrations in groundwater are stable over decades [consistent with other research (e.g., Steinmaus 2005)], justifying the use of geospatial models based on the mean arsenic concentration in individual private wells over decades to predict spatial variability of inorganic arsenic in groundwater. This was supported by a correlation analysis in a 10% sample of observed and predicted values (ρ = 0.715; 95% CI: 0.67, 0.75) (James et al. 2014).

An exposure matrix was developed to estimate each participant’s annual exposure to arsenic in drinking water. Each record included residential, employment, and school location and an estimate of the amount of water ingested and water arsenic concentration (either observed or predicted) for each participant for each year of life from birth to death or 1998, whichever came first. Three estimated exposure values were calculated for each year of the follow-up period 1984 through 1998 or CHD diagnosis, whichever came first. The three estimated exposure values—residential arsenic concentration (arsenic concentration in drinking water at residence), residential arsenic dose (residential arsenic concentration in drinking water times the amount of water consumed in liters per day), and total arsenic dose (residential arsenic dose plus workplace and/or school dose)—were each defined based on a time-weighted average (TWA). A time-weighted average (TWA) for each exposure metric was calculated by dividing cumulative per-person arsenic exposure by the number of years in each participant’s lifetime to get an annuitized exposure per year (Meliker et al. 2010).

To determine which TWA exposure estimate best approximated biologic exposure, we correlated these with speciated arsenic concentrations in historically collected urine samples (collected 1984–1991), adjusting for sex and creatinine (James et al. 2013). In brief, estimates of residential arsenic concentration (*R*^2^ = 0.37; ρ = 0.61) were the strongest correlates of the sum of the toxic urine arsenic species [As^3+^, As^5+^, dimethylarsinic acid (DMA), monomethylarsinic acid (MMA)], as opposed to estimates that included water consumption (residential dose) (*R*^2^ = 0.21; ρ = 0.46) or exposure at work or school (total dose) (*R*^2^ = 0.23; ρ = 0.48).

*Statistical analyses*. We used a Cox proportional-hazards model incorporating a robust variance estimator specific for case-cohort study designs (Barlow et al. 1999) to examine the association between TWA inorganic arsenic exposure and diagnosis of or death from CHD. We scaled the continuous arsenic exposure estimate to the interquartile range (IQR) (15 μg/L), along with other continuous covariates, similar to methods used by Lin and Huang (1995).

As described above, participants had longitudinal data from two to four study visits, including information on known risk factors for CHD. Risk factors for CHD believed to be independent of the mechanistic pathways proposed for arsenic were included in the proportional-hazards multivariate model as time-dependent covariates [lipid measurements, body mass index (BMI), physical activity, smoking, and alcohol and water consumption]. The univariate model included TWA inorganic arsenic as a continuous value scaled to the IQR (15 μg/L). Person-years and exposure were censored for CHD cases at time of diagnosis. The full model included the demographic risk factors ethnicity (white non-Hispanic/Hispanic), sex (male/female), and annual household income (high ≥ $20,000/low < $20,000); the known risk factors first-degree family history of CHD (no/yes), BMI (IQR scaled, median = 26.7, IQR = 23.8–29.3), diabetes diagnosis before CHD (no/yes); behavioral risk factors including current smoking status (no/yes), alcohol consumption (low ≤ 168 g/week/high > 168 g/week), and physical activity level (active/sedentary) (Mayer 1991); and continuous clinical risk factors including serum lipid measurements [high-density lipoprotein (HDL), triglycerides, and low-density lipoprotein (LDL) in milligrams per deciliter], hypertension (blood pressure > 140/90 or use of anti-hypertensive medicine) and folate and selenium intake (micrograms). Triglyceride and HDL levels were determined using enzymatic methods, and LDL levels were calculated using the Friedewald equation (Friedewald et al. 1972); folate and selenium intake were estimated based on 24-hr diet recall involving two- and three-dimensional visual aids for portion approximation. Nutritional analysis was based on version 14 of the Nutrition Coordinating Center’s nutrient database released in 1987 (www.ncc.umn.edu/products/database.html). Vitamin supplement use was assessed through self-report using vitamin bottle labels.

In addition, a final parsimonious model incorporating statistically significant covariates to the model based on a 10% change to the HR for TWA arsenic exposure. Known independent risk factors, including sex and family history, that were significantly associated with the outcome were maintained in all models regardless of whether or not they met statistical criteria for confounding. Covariate data were assessed at each clinic visit (up to four visits) from 1984 through 1998. The risk associated with covariates was based on the covariate value at the clinic visit before the time of the CHD event. Clinic visits assessed all behavioral and clinical values for all covariates in this analysis. Missing data occurred when participants did not attend follow-up clinic visits; however, this was a very small number (7%), so covariate values from the baseline visit were used.

We also assessed the HR for CHD across arsenic exposure groups (TWA concentration, 20–30 μg/L, 30–45 μg/L, and 45–88 μg/L relative to < 20 μg/L). The cut-points for the exposure groups are based on arsenic concentrations in past research with significant associations with CHD (Chen Y et al. 2013; Moon et al. 2013).

We assessed whether hypertension might confound the association between inorganic arsenic exposure and CHD by reanalyzing the final model with hypertension as a dichotomous covariate. Last, we completed secondary analyses to data collection methods and exposure estimates. The first was an agreement analysis on 5% of the interviewed participants (*n* = 28) that compared residential address and year reported in the interview by the participant with the residential county clerk records. We compared residence and year for the years 1955–1985 as reported by both sources. The next secondary analysis was completed in a limited cohort (*n* = 462) to confirm any association found based on residential history using mean speciated urinary arsenic concentrations (As^3+^, As^5+^, MMA, and DMA). To complete this analysis, we compared urinary arsenic concentrations between cases and noncases as a secondary analysis. We used SAS 9.2 (PROC PHREG; SAS Institute Inc, Cary, NC) for the statistical analyses. We complied with all applicable requirements of national and international regulations including approval from institutional review board, and human participants provided written informed consent before participating in the study.

## Results

This study included a cohort of 555 participants, of whom 96 were cases, for a total of 6,773 person-years of follow-up (1984 through 1998 or CHD diagnosis). The subcohort had a median age of 57 years and was 53% white non-Hispanic (Table 1). Cases were 10 years older than noncases at the baseline visit, had higher percentages of non-Hispanic whites and males, and had higher LDL and triglyceride levels; however, cases and noncases were similar with respect to family history of CHD, household income, smoking, BMI, physical activity, and consumption of alcohol and water (data not shown). The distributions of most CHD risk factors were not statistically different across arsenic exposure groups except for the low-income group, which had higher percent in the higher-exposure groups (Table 2).

**Table 1:** Baseline demographic, clinical, and behavioral characteristics of study participants with and without incident CHD during follow-up (*n *= 555).

Variable	Total subcohort (*n* = 555)
Arsenic exposure TWA (μg/L-year)
1–20	428 (77)
20–30	86 (15)
30–45	33 (6)
45–88	8 (1)
Age (years, baseline)	57 (46–64)
Ethnicity
White non-Hispanic	296 (53)
Hispanic	259 (47)
Sex
Male	267 (46)
Female	288 (54)
Income
Low	304 (47)
High	251 (53)
First-degree family history of CHD
No	439 (81)
Yes	116 (19)
BMI (*n *= 1 missing)	26.0 (23.7, 29.4)
Diabetic (diagnosed at baseline visit)
No	546 (98)
Yes	9 (2)
Current smoker
Yes	262 (48)
No	293 (52)
Alcohol (g/week)
≤ 168	528 (95)
> 168	27 (5)
Water consumption (cups/day)
< 5	225 (39)
≥ 5	330 (61)
Physical activity
Sedentary	367 (69)
Active	188 (31)
Serum LDL (mg/dL) (*n *= 10 missing)	134 (108, 163)
Serum HDL (mg/dL) (*n *= 4 missing)	46 (38, 56)
Serum triglycerides (mg/dL) (*n *= 4 missing)	145 (102, 197)
Folate (mg) (dietary assessment estimate)	257 (183, 137)
Selenium (mg) (dietary assessment estimate)	98 (89, 48)
Values are *n* (%) or median (IQR).	

**Table 2:** Baseline demographic, clinical, and behavioral risk factors for CHD across time-weighted average arsenic exposure groups (*n* = 555).

Variable	1–20 μg/L-year (*n* = 428)	20–30 μg/L-year (*n* = 86)	30–45 μg/L-year (*n* = 33)	45–88 μg/L-year (*n* = 8)
Age (years)	67 (59–73)	68 (62–73)	69 (63–73)	69 (66–75)
Ethnicity
White non-Hispanic	218 (51)	58 (67)	15 (5)	5 (63)
Hispanic	210 (49)	28 (33)	18 (54)	3 (37)
Low income (< $20,000)*	257 (60)	25 (29)	18 (55)	4 (50)
Sex
Male	208 (49)	40 (47)	15 (45)	4 (50)
Female	223 (52)	46 (53)	17 (53)	2 (25)
Family history	94 (22)	17 (20)	3 (9)	2 (25)
BMI	27.11 (23.51–29.52)	26.43 (23.42–28.40)	27.20 (25.84–29.61)	25.31 (21.02–28.22)
Diabetic (diagnosed at baseline visit)	8 (2)	0	1 (4)	0
Current smoker	208 (49)	40 (47)	11 (33)	3 (37)
Alcohol > 168 g/week	18 (4)	7 (9)	2 (6)	0
Water consumption ≥ 5 cups/day	264 (62)	39 (45)	22 (66)	5 (63)
Sedentary physical activity	291 (68)	55 (64)	18 (64)	3 (37)
Serum LDL (mg/L)	257 (104–159)	126 (102–154)	125 (107–151)	132 (101–151)
Serum HDL (mg/L)	88 (35–55)	46 (38–54)	43 (36–51)	43 (34–56)
Serum triglycerides (mg/L)	294 (98–208)	162 (138–227)	163 (138–227)	124 (87–139)
Folate (mg)	234 (158–157)	277 (235–217)	320 (253–304)	232 (176–66)
Selenium (mg)	89 (83–52)	119 (106–71)	114 (93–58)	98 (90–32)
Values are *n* (%) or median (IQR). *Statistically different in chi-square test across groups, *p *< 0.05.				

We estimated that a 15-μg/L increase in the TWA for residential inorganic arsenic water concentration was associated with a 36% higher risk for CHD (HR = 1.36; 95% CI: 1.06, 1.75 per 15 μg/L) (univariate model, Table 3). In a secondary analysis with TWA exposure categorized by four groups (univariate model, Table 3), we found a significant increase in the HR with increasing levels of arsenic exposure in a log rank test for trend (*p* = 0.0007).

**Table 3:** Cox proportional-hazards modeling results for the primary analysis of the association between CHD and TWA inorganic arsenic exposure as a continuous variable.

Variable	Univariate model HR (95% CI)	Full model HR (95% CI)	Final model HR (95% CI)
Arsenic exposure TWA (15 μg/L)^*a*^	1.36 (1.11, 1.82)	1.41 (1.09, 1.81)	1.38 (1.09, 1.78)
Female sex		0.38 (0.23, 0.64)	0.35 (0.19, 0.53)
Hispanic ethnicity		1.12 (0.70, 1.88)
Primary family member diagnosed with CHD		1.68 (0.98, 2.89)	1.75 (1.07, 2.88)
Low income		1.17 (0.67, 2.06)
Diabetic (diagnosed at baseline visit)		1.18 (0.15, 9.52)
BMI (per 5.5 kg/m^2^)^*a*^		0.81 (0.54, 1.20)
Sedentary physical activity		1.11 (0.69, 1.79)
Current smoker		1.02 (0.63, 1.65)
High alcohol consumption		1.76 (0.70, 4.41)
Low-density cholesterol (53 μg/dL)^*a*^		1.47 (1.05, 2.07)	1.40 (1.04, 1.88)
High-density cholesterol (17 μg/dL)^*a*^		0.64 (0.43, 1.01)
Triglycerides (90 μg/dL)^*a*^		0.94 (0.67, 1.34)
Folate (57 μg)^*a*^		1.00 (0.99, 1.00)
Selenium (185 μg)^*a*^		0.99 (0.99, 1.00)
Univariate model: proportional-hazards model with TWA arsenic exposure (main risk factor) as independent variable. Full model: proportional-hazards model with TWA arsenic exposure (main risk factor) and all listed variables as time dependent independent variables. Final model: proportional-hazards model with TWA arsenic exposure (main risk factor) and statistically significant covariates (independent variables). Time-dependent covariates are BMI, physical activity, smoking status, alcohol consumption, serum lipid levels, and micronutrient intake. ^***a***^IQR of subcohort at baseline.			

Estimates based on the final model also indicated a positive association with inorganic arsenic concentrations in drinking water (HR = 1.41; 95% CI: 1.09, 1.81 per 15 μg/L) (Table 3, full model). LDL and family history were significant risk factors, and being female was a protective factor. The final adjusted model (Table 3, final model) showed that time-weighted average inorganic arsenic exposure maintained an association with increased risk for CHD (HR = 1.38; 95% CI: 1.09, 1.78 per 15 μg/L) while adjusting for sex, family history of CHD, and LDL levels. When inorganic arsenic exposure was categorized, the HRs across exposure groups increased with increasing level of exposure (*p* < 0.0007) with adjustment for sex, calculated LDL, and presence of a first-degree family member with a CHD event (Table 4, final model). When hypertension was added to the final model, the association remained similar to the final model without hypertension (HR = 1.36; 95% CI: 1.06, 1.74 per 15 μg/L), suggesting that the arsenic effect on CHD is likely operating through other mechanisms.

**Table 4:** Cox proportional-hazards modeling results for the secondary analysis of the association between CHD and categorical TWA inorganic arsenic exposure.

Variable	Univariate model HR (95% CI)	Full model HR (95% CI)	Final model HR (95% CI)
Arsenic exposure TWA
1–20 μg/L-year^*a*^	1.0	1.0	1.0
20–30 μg/L-year^*b*^	1.24 (0.70, 2.31)	1.25 (0.60, 2.61)	1.23 (0.56, 2.18)
30–45 μg/L-year^*c*^	2.14 (1.22, 3.98)	2.08 (1.11, 3.92)	2.18 (1.23, 4.02)
45–88 μg/L-year^*d*^	3.12 (1.11, 9.02)	3.34 (1.15, 9.30)	3.10 (1.10, 9.11)
Female sex		0.41 (0.24, 0.69)	0.35 (0.19, 0.48)
Hispanic ethnicity		1.01 (0.62, 1.70)
Primary family member diagnosed with CHD		1.55 (0.88, 2.70)	1.63 (0.94, 2.82)
Low income		1.21 (0.71, 2.20)
Diabetic (diagnosed at baseline visit)		2.05 (0.51, 9.21)
BMI (per 5.5 kg/m^2^)		0.85 (0.59, 1.22)
Sedentary physical activity		1.12 (0.65, 1.80)
Current smoker		1.03 (0.63, 1.68)
High alcohol consumption		1.70 (0.72, 4.10)
Low-density cholesterol (53 μg/dL)^*e*^		1.46 (1.03, 2.08)	1.40 (1.02, 1.99)
Triglycerides (90 μg/dL)^*e*^		0.94 (0.65, 1.34)
High-density cholesterol (17 μg/dL)^*e*^		0.67 (0.43, 1.02)
Folate (57 μg)^*e*^		1.00 (0.99, 1.01)
Selenium (185 μg)^*e*^		1.00 (0.99, 1.03)
Univariate model: proportional-hazards model with TWA arsenic exposure (main risk factor) as independent variable. Full model: proportional-hazards model with TWA arsenic exposure (main risk factor) and all listed variables as time-dependent independent variables. Final model: proportional-hazards model with TWA arsenic exposure (main risk factor) and statistically significant covariates (independent variables). Time-dependent covariates are BMI, physical activity, smoking status, alcohol consumption, serum lipid levels, and micronutrient intake. ^***a***^Person-years of follow up = 4,806. ^***b***^Person-years of follow up = 1,335. ^***c***^Person-years of follow up = 534. ^***d***^Person-years of follow up = 98. ^***e***^IQR of subcohort at baseline.			

In the secondary analyses, we found that 73% of records matched between interview and county clerk records. We also found that cases had a statistically higher level of toxic urine arsenic species (As^3+^, As^5+^, MMA, and DMA) (μ = 20.5 μg/g creatinine in noncases vs. μ = 27.1 μg/g creatinine in cases; *p* = 0.04).

## Discussion

In this prospective study, we found that lifetime exposure to low levels of inorganic arsenic in drinking water (10–100 μg/L) was associated with increased risk for CHD. We estimated that for every 15-μg/L increase in arsenic concentration in residential drinking water, the risk for CHD increased by 38%; and across increasing levels of exposure, risk increased in a dose-dependent fashion (trend *p* = 0.0007) after adjusting for sex, family history of CHD, and serum LDL levels.

The wide spectrum of longitudinal clinical, behavioral, and demographic data in SLVDS, plus a low rate of out-migration, along with variability in inorganic arsenic exposure in the San Luis Valley, renders this region and cohort particularly suitable for this research. Inorganic arsenic in groundwater in the San Luis Valley is natural, resulting primarily from weathering and erosion of rock formations (Neely 2002), and spatial variation is due to long-term patterns of rainfall and physio-chemical conditions (Abernathy et al. 2003; Hinwood et al. 2003).

We used a thorough residential and employment history, coupled with a comprehensive spatial prediction model of groundwater concentrations of inorganic arsenic, to characterize a life-course time-weighted average arsenic exposure at the individual level. The selection of residential arsenic concentration as the exposure metric was based on a correlation analysis with speciated arsenic concentrations in historically collected urine samples from this same cohort.

One plausible mechanism for arsenic cardiotoxicity is through the creation of oxygen radicals including lipid peroxidase, which can initiate endothelial cell proliferation, function, and apoptosis, a precursor to atherosclerosis (Chen Y et al. 2009; Hirano et al. 2003; Navas-Acien et al. 2005; Pi et al. 2002; Ratnaike 2003; Santra 2000; Waalkes et al. 2000). Studies in high-arsenic areas of Asia have found increased levels of circulating reactive oxygen species such as hydrogen peroxide, hydroxyl radicals, and superoxide radicals (Yamanaka et al. 1990) and higher blood levels of lipid peroxidase (Pi et al. 2002) versus low-exposure comparison groups. Other arsenic toxicity mechanisms that have been suggested include vascular smooth muscle cell proliferation and dysfunction (Bae et al. 2008), inhibited endothelial nitric oxide synthase activity (Kao et al. 2003; Lee et al. 2003), smooth muscle cell migration (Simeonova and Luster 2004), and enhanced platelet aggregation (Lee et al. 2002).

Past research has documented an association between hypertension and inorganic arsenic exposure (Chen CJ et al. 1995, 2007; Rahman et al. 1999); a systematic review of 11 studies (Abhyankar et al. 2012) also corroborated an association between arsenic exposure in drinking water and hypertension, even at low concentrations of arsenic. These findings suggest that arsenic may be related to CHD through a pathway that includes hypertension, a known risk factor for CHD; consequently, hypertension was not included as an independent risk factor in our models. We assessed hypertension as a potential confounder and found no change in the association between CHD and arsenic exposure. Also, although the study was adequately powered to investigate risk from arsenic exposure, there may be concern that well-known risk factors for CHD, including BMI and smoking, were not associated with CHD in this small cohort; however, other known CHD risk factors (family history of CHD, serum LDL levels, and sex) were significantly associated.

Recent research has suggested that intake of folate and selenium can influence arsenic metabolism and the association between cardiovascular disease (George et al. 2013); however, in this study, folate and selenium intake levels did not significantly contribute to the hazards model nor significantly change the association. This difference in finding could be attributable to variations in estimation of micronutrient intake in this study compared with others, and therefore should be a consideration to improve measurements for future research.

There exists the possibility of misclassification bias due to the exposure estimation in the use of exposure predication models and residential history reconstruction. Although our groundwater modeled predictions were correlated with arsenic concentrations measured in urine samples (ρ = 0.63), misclassification cannot be ruled out. We also found in a limited cohort that cases had a statistically higher level of toxic urine arsenic species, suggesting that in a different metric of exposure an association between inorganic arsenic exposure and CHD also exists. Specific to the residential reconstruction, the primary method for data collection (interview) varied in response by case status (30% noncases, 45% cases) although not by exposure status, which could induce misclassification bias. We believe that misclassification bias would be small given the low migration of this population [5% migrated to the SLV as children, and only 10 participants (war veterans) lived outside of the SLV for > 6 months (< 3 years) through 1998] and the validation of clerk records and SLVDS database to complete residential history.

The exposure assessment does not include exposure resulting from ingesting contaminated food, inhalation of dust or soil, or use of tobacco products. In a comprehensive review of literature and analysis of arsenic, the Agency for Toxic Substances and Disease Registry (2007) noted that in areas of the United States where arsenic levels in drinking water are > 10 μg/L, ingestion of drinking water is the dominant source of inorganic arsenic exposure relative to the U.S. diet and inhalation through air, and therefore confirms our use of drinking water as the main source of exposure (Tao and Bolger 1999).

The exposure assessment remains limited by potential misclassification bias. Thirty-three percent of the subjects had a residential history created through records at the county clerk office because they were not available (e.g., deceased) for interview. However, the use of county clerk records was confirmed in participants who were not interviewed, where we found that data collected from county clerk records had strong agreement with self-reported residential history. For the subjects with imputed residence (*n* = 18 subjects, 126 person-years), we looked at city for the address before and after the period with missing residence and found an 89% agreement, suggesting that many residents may move houses, but not necessarily out of the city or out of the San Luis Valley. Self-reported estimates of lifetime residential, employment, and schooling locations and duration, and number of cups of water consumed per day also are likely limited by inaccuracies leading to misclassification bias which could bias the findings.

Another limitation is that the arsenic exposure estimates were included in the proportional-hazards model under the assumption of no error. Past research has incorporated bootstrap methods to incorporate an error term for the estimate in logistic regression models, but to date has not been done in a proportional-hazards model. A future step would be to develop the statistical methodology for incorporating the error term associated with the predicted arsenic exposure into the proportional-hazards model.

A recent study from a high arsenic area reported an HR for CHD of 1.22 (95% CI: 0.65, 2.32) at arsenic levels of 12.1–62.0 μg/L, similar to levels found in the SLV, while controlling for known CHD risk factors (Chen Y et al. 2011). Using a comprehensive exposure assessment, our study found consistent results at lower levels (1–100 μg/L), with a proportional-hazards ratio of 1.75 for exposure levels from 30 to 45 μg/L relative to < 20 μg/L. Our findings plus those by Moon et al. (2013), who identified a similar association between incident cardiovascular disease and exposure to low to moderate arsenic levels with exposure defined through urine biomarkers, indicate that a dose–response relationship between arsenic and CHD exists at levels of arsenic that are not uncommon in many areas.

Inorganic arsenic exposure in drinking water has been identified as a cardiotoxic element at concentrations seen in drinking water supplies around the world which strengthens the importance of ensuring public water supplies meet the U.S. EPA maximum contaminant level (MCL) of 10 μg/L (U.S. EPA 1998). Currently, many areas of the United States have levels above the U.S. EPA MCL, including western states of Nevada, Colorado, and Arizona; Midwest areas including Michigan; and Northeastern areas of New Hampshire, Maine, and Connecticut (U.S. EPA 2011).

In conclusion, we observed an association between CHD risk and inorganic arsenic exposure in a chronic low-level arsenic area in the southwestern United States. Because arsenic in drinking water remains a common exposure in the United States, the risk of CHD should provide motivation to public health officials to bring drinking water levels into compliance (< 10 μg/L) and to conduct further research to elucidate the role of arsenic in the pathobiology of CHD.
